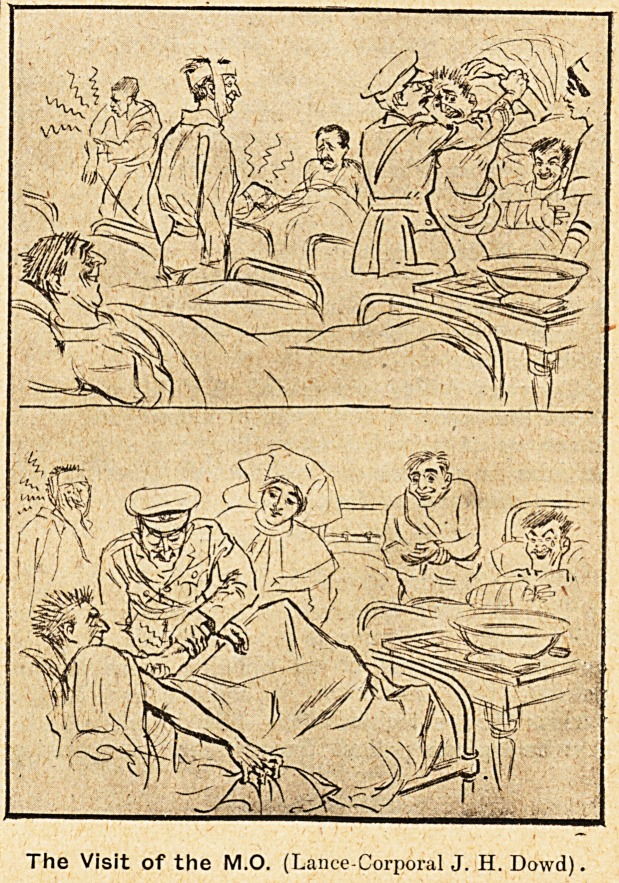# In a Territorial Hospital

**Published:** 1917-12-22

**Authors:** 


### In a Territorial Hospital.

**The Visit of the M.O.** (Lance-Corporal J. H. Dowd).

## Figures and Tables

**Figure f1:**